# From Research to Practice: Challenges to Implementing National Diabetes Guidelines With Five Community Health Centers on the U.S.-Mexico Border^1^

**Published:** 2004-12-15

**Authors:** Kenneth A Schachter, Stuart J Cohen

**Affiliations:** Mel and Enid Zuckerman Arizona College of Public Health; Mel and Enid Zuckerman Arizona College of Public Health, Tucson, Ariz

## Abstract

**Background:**

Given the dramatic increase in type 2 diabetes in the United States, the development of effective strategies to prevent and control this potentially devastating illness is more important than ever. In the Southwest, diabetes is a far too common and rapidly growing problem among Mexican Americans living near the U.S.-Mexico border. A project designed to address this problem enabled faculty from the University of Arizona to work with community health centers to evaluate and improve diabetes care in border communities.

**Context:**

This project was a component of the Border Health Strategic Initiative (*Border Health ¡SI!) *and Racial and Ethnic Approaches to Community Health 2010 (REACH 2010), both funded by the Centers for Disease Control and Prevention. University of Arizona faculty worked in partnership with five community health centers funded by the Health Resources and Services Administration. The goal of the faculty was to assist the community health centers with 1) development of measures of diabetes care based on national clinical practice guidelines, 2) identification of gaps in care based on those measures, and 3) implementation of strategies for closing those gaps.

**Methods:**

All five centers prioritized their top four or five indicators of diabetes care (e.g., annual dilated eye examination). Different community health centers selected different indicators. Baseline medical record audits were performed using the chosen indicators. Individual results were shared confidentially with providers; overall center results were shared and discussed with providers and staff.

**Consequences:**

Each clinic chose its own strategies for closing gaps in care. At one-year follow-up, there was evidence of improvement for the majority of indicators in all community health centers. However, some gaps remained. Of the three community health centers having a second-year evaluation, two maintained or increased the improvements made, but one lost ground.

**Interpretation:**

Our experience with these five border clinics was that translating guidelines into practice is easier said than done. Factors that favored success included an onsite champion, staff buy-in, a willingness to see systems change, and the availability of additional resources, particularly for chart reviews.

## Background

Between 1990 and 2000, the Mexican population in the United States increased by 52.9%, from 13.5 million to 20.6 million. By 2050, it is estimated that there will be 97 million Hispanic Americans in the United States, comprising about one quarter of the total population (1). In 2000, more than 43% of Hispanics lived in the West. Half of all Hispanics lived in just two states, California and Texas. The largest Mexican populations were in California, Texas, Illinois, and Arizona. In the three border states (California, Texas, and Arizona), Hispanics were in the majority in 50 counties along the U.S.-Mexico border (2).

Diabetes was the sixth leading cause of death in the United States in the year 2000. More than 17 million Americans (about 2 million Hispanic Americans) have been diagnosed with diabetes, and approximately 1 million more individuals, aged 20 years and older, are diagnosed with diabetes each year (3). At any given age, Mexican Americans are twice as likely to have type 2 diabetes as non-Hispanic whites (4).

In 2002, researchers at the Centers for Disease Control and Prevention (CDC) developed a Diabetes Report Card to examine quality of diabetes care in the United States during the 1990s based on nationally accepted guidelines for care. Their research revealed that 18% of persons with diabetes aged 18 to 75 years had very poor glycemic control (HbA1c values >9.5%) and that 34% had elevated blood pressures (⩾140/90 mm Hg). Left untreated or inadequately treated, both conditions will lead to increased morbidity and mortality (3). Additionally, 45% of patients with diabetes had not had a foot examination during the previous year and 37% had not had a dilated eye exam (3).

In a September 2004 report on the quality of care among its member health plans, The National Committee for Quality Assurance (NCQA) asserts that many Americans do not receive adequate preventive care and/or care for chronic conditions like diabetes and hypertension. It further asserts that the gap between the less-than-optimal health care that most Americans receive and the care that some receive from the best health plans results in anywhere between 42,000 and 79,000 premature deaths per year (5).

There is increasing evidence of programs and treatment strategies that are effective in controlling diabetes and preventing its complications; however, the translation of that evidence into medical practice continues to lag. The Institute of Medicine estimates that the time between the discovery of an effective treatment and its incorporation into routine care is as long as 17 years and that more than 50% of patients with such common conditions as diabetes, hypertension, tobacco addiction, hyperlipidemia, congestive heart failure, asthma, depression, and chronic atrial fibrillation are inadequately managed (6).

## Context

Community Health Centers (CHCs) first received federal funding as part of the War on Poverty in the mid-1960s. Approximately 100 CHCs (known at the time as neighborhood health centers) had been funded under the Economic Opportunity Act (EOA) by the early 1970s. These centers made culturally appropriate health care accessible to many low-income families. In 1969, the Public Health Service (PHS) also began funding neighborhood health centers. When the EOA was phased out in the early 1970s, centers previously supported under it were transferred to the Public Health Service (PHS). Today, CHC funding is authorized under section 330 of the PHS Act through the Health Resources & Services Administration (HRSA). CHCs exist in areas where economic, geographic, or cultural barriers limit access to primary health care. Their mission is to provide family-oriented, primary, and preventive health care services for people living in rural and urban medically underserved communities (7).

This paper describes our experiences as university faculty with the primary care providers (medical doctors, doctors of osteopathy, nurse practitioners, and physician assistants) and support staff in five community health centers participating in two federally funded projects, The Border Health Strategic Initiative (*Border Health ¡SI!*) and Racial and Ethnic Approaches to Community Health 2010 (REACH 2010). All five centers are located in the United States near the U.S.-Mexico border and care for large Hispanic populations with a high prevalence of diabetes. The border region includes four states in the United States (Arizona, California, New Mexico, and Texas) and six states in Mexico (Baja California, Chihuahua, Coahuila, Nuevo Leon, Sonora, and Tamaulipas).

In 2000, approximately 11.3 million people lived on both sides of the border — 6,268,107 individuals on the U.S. side, and 5,054,516 on the Mexico side (8). The U.S. side is approximately 70% Hispanic, with a higher population growth rate (1.8%) than the national rate (0.9%). Five of the seven poorest counties in the United States are on the border, and more than 30% of Hispanics living on the border are uninsured (9).

## Methods

In all five centers, we met with the medical directors and other clinical management staff. First, we explained the project and our approach. Next, we worked with medical directors to develop target guidelines, or indicators. We used an "indicators of care" form to help them identify five aspects of diabetes care they most valued. We limited the medical directors to five aspects out of concern that they might try to accomplish too much, too soon. One center tracked only four indicators after a mid-course change. We encouraged the medical directors to involve their medical and other health care staff in this selection process, and we had conversations with the medical directors and/or their staff about their choices, especially when there was not good evidence to support a selected intervention or intervention frequency. However, the centers' choices always prevailed, even if they selected a measure for which there was not good evidence. Some medical directors were more interested than others, some had more participatory management styles than others, and some delegated more than others. We adapted our procedures to the local characteristics of each center. Each CHC was given the option of having record reviews performed and reported only at the center level or at both the center and provider levels, with the provider-level results being shared confidentially with individual providers. All CHCs opted for both provider and center-level results.

Primary care providers for each clinic were eligible to have their patients' charts included in the record review if the provider had practiced at the CHC for the 12 months prior to the review. Not all providers at each CHC participated in this project. In some CHCs with multiple offices, participation was limited to one site. In addition, while many of the same providers participated from the beginning to the end of the project, staff turnover led to changes in those individuals being reviewed from one year to the next. As planned, we completed three rounds of data collection (baseline, one-year follow-up, and two-year follow-up) in three clinics and two rounds (baseline, one-year follow-up) in the other two. In 2001, we audited 22 providers' records from five participating CHCs; in 2002, 19 providers' records from five CHCs; and in 2003, nine providers' records from three CHCs.

After indicators were selected, we developed indicator-specific training manuals for medical records reviewers. The manual was designed to allow us to train staff with little or no medical records review experience and to serve as a reference for questions reviewers might have during the review process. Reviewers were instructed to begin auditing charts soon after their training was completed. Charts were randomly selected from up-to-date listings of patients with diabetes assigned to each primary provider. For a chart to be eligible, the patient had to be at least 18 years of age, had to have a diagnosis of diabetes based on Current Procedural Terminology 250.XX codes, and had to have visited his/her primary provider at least once during the 12 months under review. If there were multiple visits during the 12 months, the primary provider had to have seen the patient for a majority of those visits.

Obtaining provider-level data required a larger sample than would have been necessary for clinic-level data. Evidence from prior studies indicated that 12 to 15 charts per provider are needed to obtain a stable estimate of provider performance while imposing the lowest possible burden on center staff. To ensure and improve the reliability of our reviewers, we used two reviewers in every center for each review cycle, asking that they assign a primary and secondary reviewer for each provider's records. The primary reviewer reviewed all of that provider's records. The secondary reviewer randomly selected and reviewed two of that provider's records while avoiding discussing them with the primary reviewer and/or viewing his/her audit results. The secondary reviewer was then instructed to compare both reviews and mark all inconsistencies on the secondary review form. Both reviewers were then asked to review the disagreements and, where indicated, correct any mistakes on the primary review form. The primary review forms were used to calculate the level of compliance with selected indicators. The marked secondary review forms, which showed primary and secondary reviewer errors, were used to calculate interrater agreement. Our goal was to achieve interrater agreement of greater than or equal to 90%. We missed that mark only twice in a total of thirteen reviews. The chart selection process was repeated for each round of reviews. These cross-sectional samples included only those randomly selected patients who met eligibility criteria for that year.

Following the initial reviews, we met with the medical and key office staff to present a table showing baseline center-level results. All participants received their CHC's results. Each individual provider also received a table in a sealed envelope comparing his/her results to center results. We promised a repeat audit in about 12 months.

We returned to reaudit charts, as promised, in approximately 12 months for two centers and in both 12 and 24 months for three centers. After each audit, we presented our results to the medical staff and discussed strategies for further improvement. In the satellite center that showed the least improvement at year one, we changed some indicators at the request of the primary provider, who had different priorities for diabetes care and had not been able to participate in the initial selection process.

## Consequences

For most indicators, overall center performance was higher at the one-year assessment than it was at baseline. In addition, two of the three CHCs having year-two assessments showed generally improved results from year one. The third center's year-two results showed worsening performance in most areas compared with year one. Given the cross-sectional nature of the samples, these results should be interpreted with caution.

Only two of the five centers prioritized the same five diabetes indicators, and only two indicators were selected by all five CHCs — namely, annual assessment of urine for microalbuminuria and HbA1c testing. For HbA1c testing, there were differences in the desired frequency, with some CHCs wanting at least two HbA1c tests per year and others wanting three tests per year. Factors that seemed to influence indicator selection and adherence included whether the selection was based on consensus or made by the medical director; provider training, experience, and beliefs; and CHC staff and organizational issues. In one CHC where the medical director chose the indicators, we later revised them midstream to reflect the priorities of a physician who had not been involved in the initial process and was the sole CHC physician participant in our initiative. From this experience and others, we learned that it was important to recognize and address local issues that could adversely affect indicator selection and/or staff buy-in and participation.

All five of our CHCs used paper records. While there is evidence that provider reminder systems such as diabetes flow sheets helped improve diabetes care, not all of our centers used them (10,11). Some were understandably resistant to adding yet another flow sheet to their already complicated charts. One CHC already had incorporated its diabetes measures into its adult health maintenance flow sheet. The majority of its patients did not have diabetes, and providers were only infrequently using that portion of the flow sheet. After some discussion, we arrived at the solution of placing colored stickers inside the charts on the adult health maintenance/diabetes flow sheets of their patients with diabetes. This change resulted in improved recognition of patients with diabetes and improved performance on the indicators. The use of flow sheets, in general, was associated with improved recognition and performance.

Any new initiative dependent on the participation of providers must compete with many other demands on their time during usual patient encounters (e.g., patient expectations and requests, professional concerns, diverse and sometimes conflicting practice guidelines and prevention recommendations, local and national initiatives, interruptions, emergencies). For example, even though four of our five partner CHCs were participating in the HRSA/CDC Diabetes Collaborative — whose members agreed to adopt local shared quality-improvement measures consistent with national guidelines — the level of participation still varied considerably from site to site. This taught us that participation in other diabetes programs was no guarantee of success.

In most centers, providers reacted to our initial presentation of results with disbelief, as both their individual and CHC levels of compliance were typically lower than they expected. During our meeting, they appeared to be comparing their results with center results and sometimes with another provider's results. We addressed the skepticism in several ways. First, we described our methods during our presentation (i.e., the comprehensiveness of the chart reviews, the use of two reviewers for quality control, the levels of interrater reliability). Second, we also asked the reviewers, who could be project and/or local office staff, to be present to respond to any questions. Third, we put the results in context by comparing each center's results with available national statistics that were typically about the same or worse. Generally, these strategies overcame barriers to acceptance, and we were able to move on to a more substantive discussion on what steps could be taken for improvement. We then facilitated discussions on what behavioral and structural changes providers could make as a staff to improve their results, and we offered technical assistance, such as help with the development of flow sheets or telephone consultation. By the end of the meeting, centers had usually developed a tentative plan for improvement. From this, we concluded that while obtaining provider-level data was more work, it generated a healthy interest and sense of competition among participants.

Given how busy providers often are, we looked for other ways to improve care. When feasible, we recommended implementing measures via "systems change" as an alternative to assigning a new responsibility to already overburdened providers. In one center, the medical director agreed with our recommendation that medical assistants take more responsibility for charting and ordering certain diabetes screening tests under standing orders, such as annual urine testing for microalbumin, annual lipid panel, and periodic HbA1c testing. We conducted a special training session for those staff. However, it took several visits before we noticed a change, and we were not confident that it would persist. We learned from this and other experiences that systems change at the practice level can be quite difficult to achieve and sustain.

## Interpretation

Despite the many competing demands on CHCs, our project did achieve some success, and we believe that it was worthwhile. We helped the CHCs focus on interventions that matter but are sometimes neglected. Our CHCs often chose indicators based on national guidelines. They were motivated to review and, in many cases, improve their performances, thereby closing the gap that exists between research and practice. Three CHCs elected to continue beyond their original three-year commitment. For the CHCs that chose not to continue after their initial commitment, the availability of resources, particularly for medical audits, was an important issue.

We want to be careful about generalizing, since we worked with only five CHCs and no two were alike. Further, as consultants, we were not always privy to the activities and interventions that took place between our visits. Nonetheless, we observed that six factors were most important to overall success in our initiative: 1) the presence of an onsite champion, 2) broad staff and managerial support and participation, 3) the willingness of providers to delegate authority to ancillary staff via standing orders for routine tasks and testing, 4) the use of flow sheets, 5) the presence of a full-service diabetes clinic, and 6) access to provider-level data. In our experience, these are the factors that most favor success. We hope that these observations will prove useful to those contemplating similar initiatives.
